# Gerobiotics and neuroprotection: effects on the gut-brain axis in age-related neurodegenerative diseases

**DOI:** 10.3389/fnagi.2026.1814234

**Published:** 2026-05-28

**Authors:** Betul Kocaadam-Bozkurt, Serkan Aslan, Osman Bozkurt, Mahmut Bodur, Duygu Agagündüz, Ferenc Budán

**Affiliations:** 1 Department of Nutrition and Dietetics, Faculty of Health Sciences, Erzurum Technical University, Erzurum, Türkiye; 2 Department of Nutrition and Dietetics, Kulu Faculty of Health Sciences, Selcuk University, Konya, Türkiye; 3 Department of Nutrition and Dietetics, Faculty of Health Sciences, Ankara University, Ankara, Türkiye; 4 Department of Nutrition and Dietetics, Faculty of Health Sciences, Gazi University, Ankara, Türkiye; 5 Institute of Physiology, University of Pécs, Medical School, Pécs, Hungary

**Keywords:** aging, gerobiotics, gut-brain axis, microbiome, neuroprotection

## Abstract

As the global population ages, effective strategies to attenuate or prevent neurodegenerative processes are becoming increasingly important. Gerobiotics, an emerging class of probiotic strains and their derived postbiotics, are considered promising geroprotective agents because of their potential to target fundamental mechanisms of aging, modulate the gut–brain axis, and attenuate age-related cognitive and functional decline. This review aims to synthesize existing evidence from preclinical and clinical studies on the neuroprotective effects of gerobiotics, with particular emphasis on ageing-related changes in gut microbiota composition, systemic inflammation, and the pathophysiology of neurodegenerative diseases. Preclinical animal studies show that gerobiotics ameliorate memory impairment, preserve synaptic integrity, and attenuate neuroinflammation. Furthermore, clinical results suggest improvements in cognitive performance, mood regulation, and gastrointestinal function, particularly in the early stages of neurodegenerative disorders and among individuals with mild cognitive impairment. The microbiota–gut–brain axis has emerged as a relevant therapeutic target, with gerobiotic supplementation representing a multidimensional approach to support healthy cognitive ageing and counteract neurodegenerative processes. The underlying mechanisms, manifested mostly through modulation of microbial metabolites, include the restoration of intestinal and blood–brain barrier integrity, the reduction of neuroinflammation, the enhancement of neurotrophic factors, and the modulation of immunological pathways. Although current evidence is promising, heterogeneity in probiotic strains, dosages, and study designs indicates the need for further rigorous investigation. Further well-designed, large-scale clinical studies are required to establish efficacy, optimize intervention protocols, and support the translation of gerobiotics into evidence-based clinical practice for the prevention and management of age-related neurodegenerative diseases.

## Graphical abstract

**Figure:**
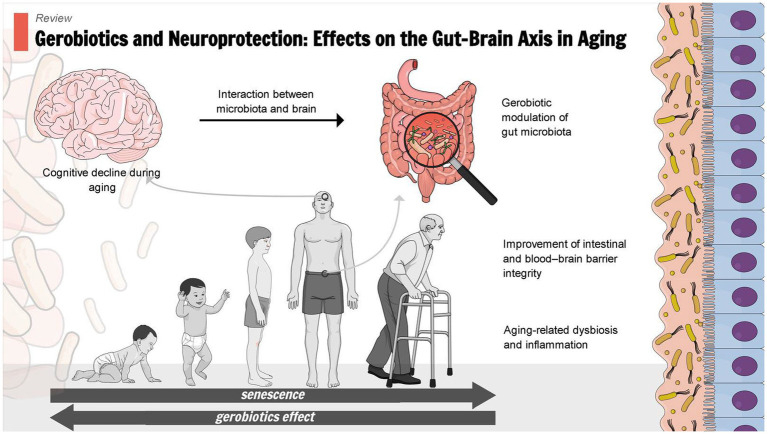

## 1 Introduction

### 1.1 Gerobiotics: definition and importance

The average lifespan of people has been increasing over the years. While life expectancy was 50 years in the 1960s, it reached 73 years in 2023 (World Bank, 2026). On the other hand, in developed countries like Italy, Spain, and Japan, this figure has reached as high as 84 years. Life expectancy has increased due to advances in healthcare, nutrition, and living conditions. The World Health Organization (WHO) predicts that between 2015 and 2050, the global population over 60 will increase from 12 to 22%. This situation brings with it many aging-related health problems (World Health Organization, 2025).

Several factors, including unhealthy dietary patterns, inappropriate antibiotic use, other pharmacological interventions, and exposure to pathogens, can adversely affect the gut microbiota. When these disturbances exceed the adaptive capacity of the gut microbiota, they may lead to dysbiosis (Cristofori et al., 2021; Caputi et al., 2017). Given the close interaction of the gut microbiota with neurological, metabolic, and immune pathways, dysbiosis may contribute to the development of neurodegenerative diseases (Philip Mani et al., 2024).

**Figure 1:**
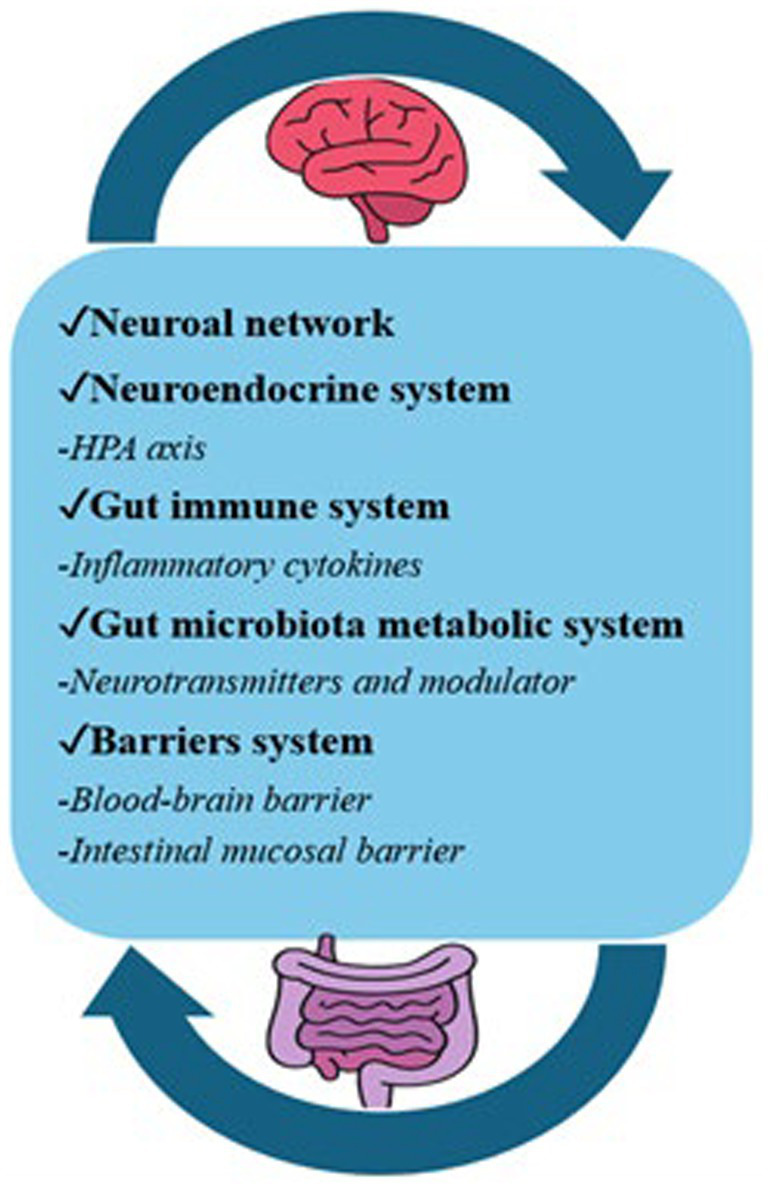
Overview of the gut–brain axis communication pathways. This figure illustrates the bidirectional communication between the gut microbiota and the central nervous system through major gut–brain axis pathways, including neural signaling via the vagus nerve and enteric nervous system. These interactions provide the basis for broader endocrine, immune, and metabolic communication involved in the regulation of brain function.

There is a strong relationship between the microbiota and the brain, which is regulated through various pathways and is related to many metabolic processes (Figure 1; Alsegiani and Shah, 2022). The microbiota can interact with the brain through two neuroanatomical pathways. The first neuroanatomical pathway connects the gut and brain via the autonomic nervous system and the vagus nerve (Bonaz et al., 2017). The second pathway facilitates bidirectional communication with the enteric nervous system in the gut and the autonomic nervous system and the vagus nerve in the spinal cord (Furness, 2012).

There are many methods for slowing down the aging process and treating and preventing health problems that arise with aging. In this context, the effectiveness of probiotics has recently gained increasing importance, and studies have highlighted their potential benefits (Bashir et al., 2024; Ghassab et al., 2024; Bulacios et al., 2023; Lee et al., 2019; Cristofori et al., 2021). According to the International Scientific Association for Probiotics and Prebiotics (ISAPP), probiotics are live microorganisms that provide health benefits to the host when administered in sufficient amounts, while prebiotics are selectively utilized substrates that beneficially modulate the host microbiota (Gibson et al., 2017). More recently, ISAPP has defined postbiotics as preparations composed of inanimate microorganisms and/or their components that exert health-promoting effects on the host. Importantly, this concept includes non-viable microbial cells or their structural components, with or without metabolites, provided that these constituents contribute to the demonstrated biological benefit (Salminen et al., 2021). Gerobiotics have been proposed as an emerging class of probiotic strains and/or their derived postbiotics that target key biological mechanisms of aging, with the potential to delay age-associated functional decline and promote healthy lifespan extension (Tsai et al., 2021; Ağagündüz et al., 2022).

Gerobiotics have the ability to go beyond intestinal health and provide potential benefits on genomic instability, telomere shortening, epigenetic changes, mitochondrial dysfunction, and aging processes (López-Otín et al., 2013). Probiotic strains such as *B. infantis* ATCC15697*, L. fermentum* MBC2*, B. longum BB68, L. paracasei* PS23*, L. gasseri* SBT2055*, L. brevis OW38, and L. paracasei* PS23 are considered to be in the gerobiotic class (Ağagündüz et al., 2022). Gerobiotic strains such as *Lactobacillus fermentum* DR9 have potential benefits on telomere attrition, which is a marker of aging, and *Lactobacillus paracasei* PS23 and *Bifidobacterium breve* B-3 strains have potential benefits on mitochondrial dysfunction (Chen et al., 2019; Toda et al., 2020; Lew et al., 2019).

### 1.2 Aging, microbiota, and neuroinflammation

**Figure 2:**
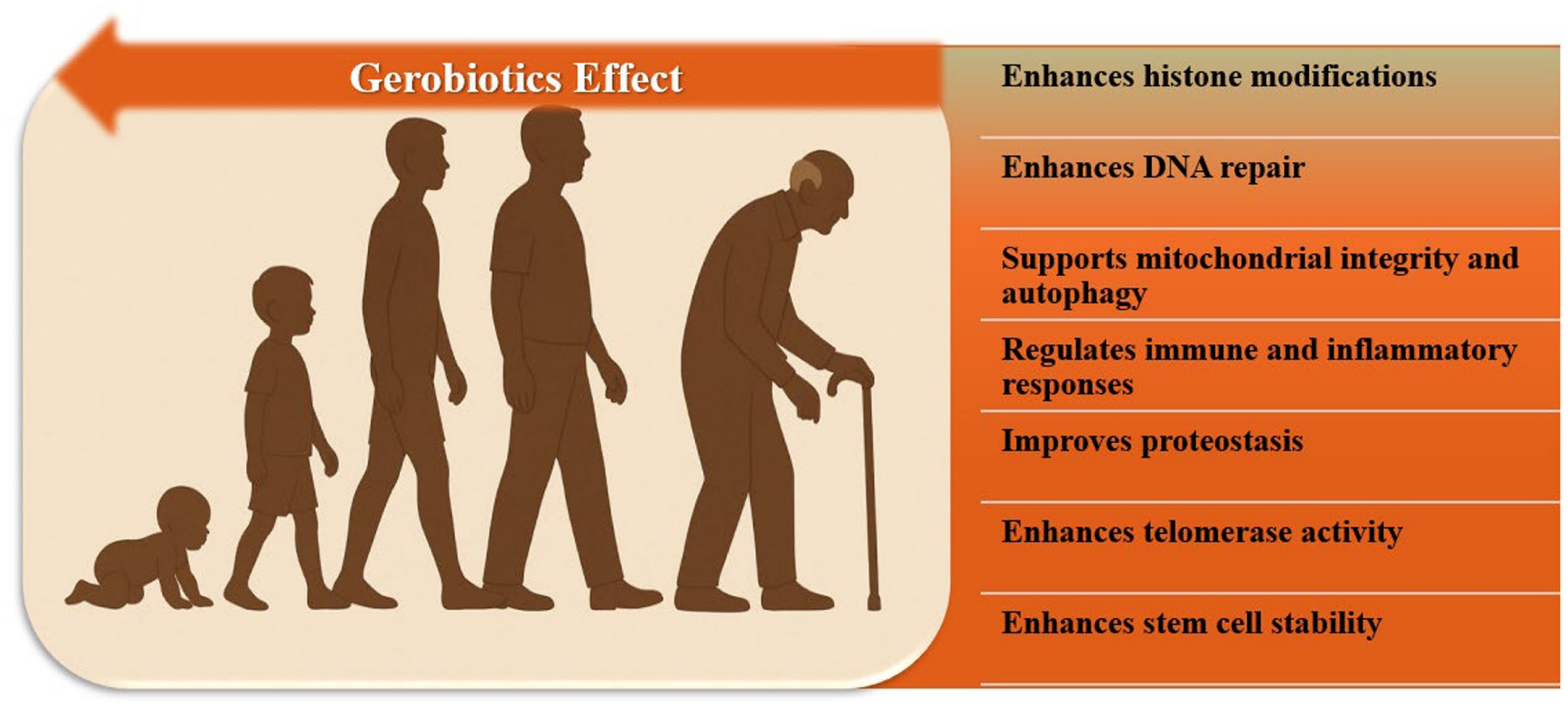
Potential effects of gerobiotics on aging-related biological processes. This figure illustrates the potential effects of gerobiotics on key biological processes associated with aging, including histone modification, DNA repair, mitochondrial integrity and autophagy, immune and inflammatory regulation, proteostasis, telomerase activity, and stem cell stability. Through these mechanisms, gerobiotics may contribute to the maintenance of cellular homeostasis and the delay of age-related functional decline.

The aging process involves a gradual decline in the body’s physiological functions. This process primarily involves DNA damage, shortening of telomere length, mitochondrial dysfunction, lipid peroxidation, and protein oxidative modification (Yang et al., 2024). Gerobiotics may influence multiple hallmarks of aging, including oxidative stress, mitochondrial dysfunction, and chronic inflammation, as illustrated in Figure 2 (Mahamud et al., 2025). Hence, it has been reported that the changes in physiological processes and increased catabolic processes with age can be slowed by the potential effects of gerobiotics (Jayanama and Theou, 2020).

The central nervous system (CNS) is also affected by aging, which is closely related to neuroinflammation (Hou et al., 2019; Pai, 2024; Soraci et al., 2024). The most common neurodegenerative diseases encountered by the elderly are Alzheimer’s disease (AD), Huntington’s disease (HD), and Parkinson’s disease (PD; Hou et al., 2019). It is known that the aging-related deactivation of immune system cells leads to increased neuroinflammation and neuronal dysfunction in Alzheimer’s, Parkinson’s, and multiple sclerosis diseases (Kritsilis et al., 2018; Plantone et al., 2023; Hou et al., 2019).

Inflammation that develops in brain tissue is a protective reaction mechanism that the body creates to heal or remove damaged tissues, an infection environment, toxins from the body, and to ensure homeostasis in the brain (Kulkarni et al., 2016; Shi et al., 2023). Aging leads to damage and imbalances in the immune system. Aging is associated with increased pro-inflammatory mediators contributing to neuroinflammation (Teissier et al., 2022). The potential anti-inflammatory and immunomodulatory actions of gerobiotics are addressed in detail in Section 4.

### 1.3 The gut-brain axis and neurodegenerative diseases

Neurodegenerative diseases comprise a variety of disorders affecting CNS. These progressive diseases cause a gradual decline in daily activities (such as cognitive function and mobility; Sirin et al., 2023). The gut–brain axis enables bidirectional physiological communication between the brain and the gastrointestinal system. The gut-brain axis is also crucial in a healthy neurological system and aging (Tsai et al., 2021). An imbalance in the gut microbiota negatively impacts the gut-brain axis, increasing the incidence of neurodegenerative diseases (Maiuolo et al., 2021; Madison and Kiecolt-Glaser, 2021).

**Figure 3:**
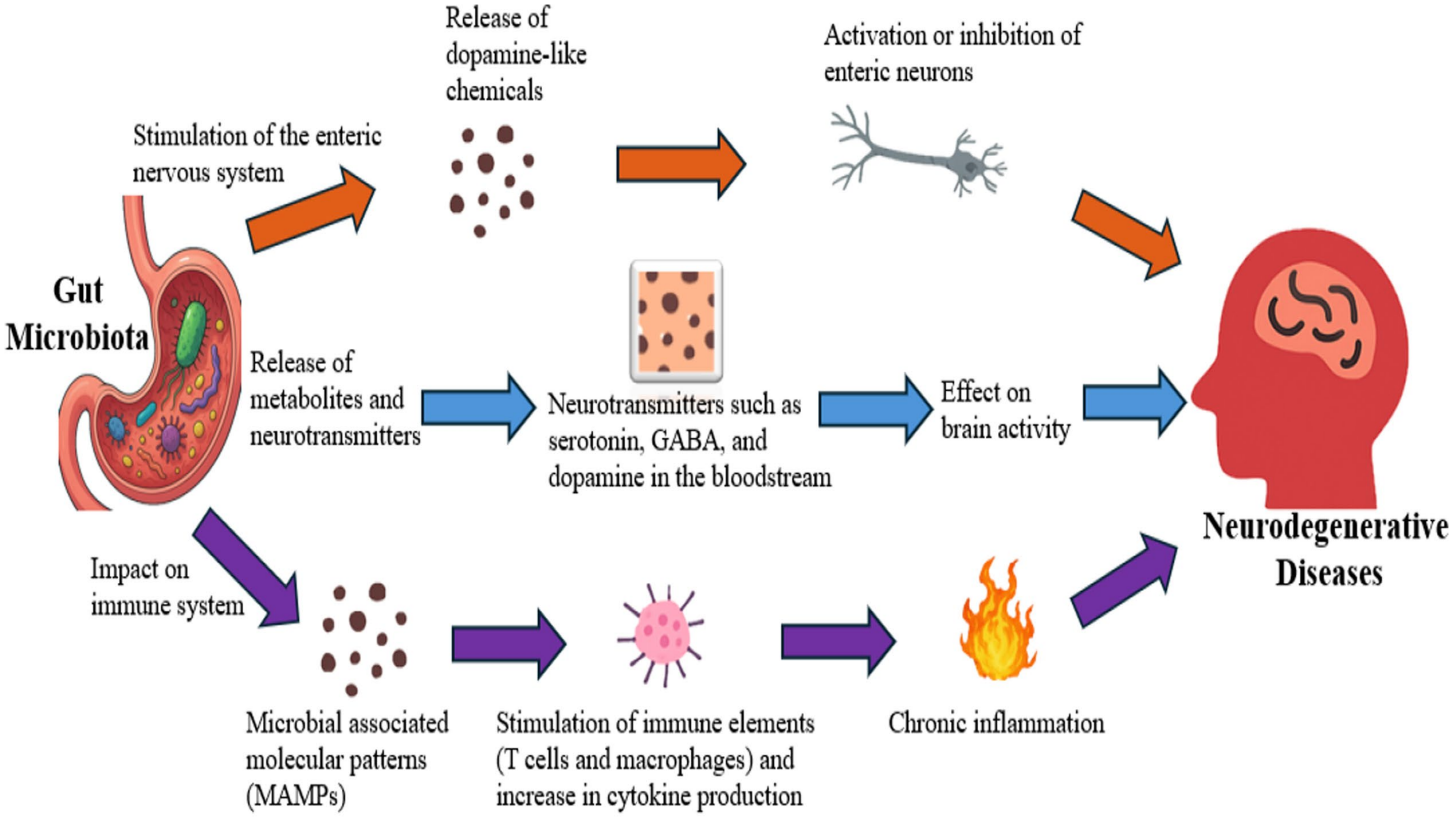
Role of gut microbiota in neurodegenerative diseases. This figure illustrates the pathways through which gut microbiota may influence brain function and contribute to neurodegenerative diseases. Microbial activity affects the central nervous system via neural signaling (enteric nervous system and neuronal activation), metabolic pathways involving neurotransmitters such as serotonin, GABA, and dopamine, and immune mechanisms including microbial-associated molecular patterns (MAMPs), activation of immune cells, and cytokine production. These interconnected pathways may lead to chronic inflammation, altered brain activity, and increased risk of neurodegenerative disorders.

Neurodegenerative diseases may arise through multiple microbiota-related pathways, including neural, immune, and metabolic mechanisms, as summarized in Figure 3 (Philip Mani et al., 2024).

It has been emphasized that outer membrane vesicles (OMVs) secreted by intestinal bacteria may have effects that can lead to and exacerbate PD. OMVs released from intestinal bacteria are a physiological mechanism that contains numerous microbial-associated molecular patterns (MAMPs) and are used to support immune responses (stimulation of T Cells and macrophages). Considering their presence in the bloodstream and their potential to cross the blood–brain barrier (BBB), they are associated with neurodegenerative diseases, especially Parkinson’s (Koukoulis et al., 2024). However, the available evidence linking bacterial outer membrane vesicles to neurodegenerative processes is still largely derived from experimental and preclinical studies, and their clinical significance in humans remains to be fully established.

There is a significant relationship between gut bacteria and the dopaminergic pathway in the brain. The microbiota plays a role in regulating dopaminergic signals (Hamamah et al., 2022). Increases or decreases in dopamine receptor expression in the striatum appear to be influenced by changes in the microbiota. Certain bacterial genera (such as *Clostridium, Bacteroides, Prevotella, Lactobacillus, Bifidobacterium, Enterococcus,* and *Ruminococcus*) have been reported to regulate receptors, transporters, and specific targets of the dopaminergic pathway (Hartstra et al., 2020; Cheon et al., 2021; Villageliú and Lyte, 2018; Cao et al., 2019).

A study examining the fecal microbiota of 161 elderly individuals reported that the microbiota composition was significantly different from that of younger adults (Claesson et al., 2011). Another study found that aging affected *Prevotella* and related genera the most. However, a decrease in *Clostridium cluster XIVa*, including *Roseburia* and *Ruminococcus*, which produce the short-chain fatty acid (SCFA) butyrate, was observed (Mäkivuokko et al., 2010).

Improving the microbiota composition in older individuals holds promise for preventing and reducing age-related neurodegenerative diseases (Hutchinson et al., 2021). Oxidative stress and inflammation play a crucial role in the development of neurodegenerative diseases (Ren and Léveillard, 2022). In one study, when Alzheimer’s patients administered probiotic milk (that contained *L. acidophilus, L. casei, L. fermentum,* and *Bifidobacterium bifidum*) for 12 weeks, improvements were observed in cognitive function tests compared to the control group. In addition, C-reactive protein (CRP) and malondialdehyde (MDA), which are markers of inflammation, were found to decrease (Akbari et al., 2016). Another study found that gerobiotics (*Limosilactobacillus fermentum*) improved memory and cognitive functions and increased GABA synthesis, vital for cognitive function, in individuals over 60 years of age (Handajani et al., 2022).

## 2 Microbiota-gut-brain axis and aging

### 2.1 Age-related changes in gut microbiota

#### 2.1.1 Structural and functional shifts in the aging microbiota

The ageing process leads to significant structural and functional changes in the gut microbiota, characterized by a decrease in microbial diversity, a reduction in beneficial taxa and an increase in potentially proinflammatory microorganisms (O'Toole and Jeffery, 2015). Research shows that the composition of the gut microbiota changes with age, with a decline in beneficial bacteria such as *Bifidobacterium* and *Lactobacillus,* and an increase in potentially harmful Gram-negative bacteria, including *Proteobacteria* and *Enterobacteriaceae* (Borrego-Ruiz and Borrego, 2024; Strasser et al., 2021). Furthermore, a decline in the *Firmicutes/Bacteroidetes* ratio and increased interindividual variability in microbiota composition are commonly observed among elderly individuals (O'Toole and Jeffery, 2015). Additionally, centenarians seem to preserve or perhaps enhance health-related taxa as *Akkermansia muciniphila* and *Christensenellaceae*, implying a microbiome composition that might support lifespan and healthy aging (Ma et al., 2023).

In addition to compositional shifts, the process of ageing has been associated with functional changes in the microbiome. SCFA production, particularly butyrate, declines significantly, impairing epithelial barrier integrity, modulating immune responses, and contributing to systemic low-grade inflammation (Shi et al., 2021). Reduced fiber intake is common among older adults, further promoting this dysbiosis by diminishing SCFA-producing bacteria and promoting gut-brain axis dysfunction (Shi et al., 2021).

Recent results show that gut microbiota alterations during aging more accurately reflect biological rather than chronological aging. Individuals experiencing frailty, managing multiple chronic diseases, or living in institutional care settings often show distinct patterns of gut dysbiosis, marked by an overgrowth of pro-inflammatory microbial species and a decline in microbial diversity (Singh S. et al., 2024). These microbial changes are closely associated with increased intestinal permeability, elevated systemic inflammation, and impaired metabolic regulation, highlighting the fundamental role of the gut microbiota in the physiological processes of aging.

Both experimental and clinical studies highlight the therapeutic promise of modulating the gut microbiota. Approaches such as fecal microbiota transplantation (FMT) from young to aged individuals or supplementation with *Akkermansia muciniphila* have been shown to partially restore microbial diversity, increase the production of SCFAs, and mitigate systemic dysfunctions linked to aging (Liu et al., 2021; Ma et al., 2023). However, it is important to recognize that environmental factors, including diet, medication use, and physical activity, play a significant role in shaping the gut microbiota throughout the aging process, emphasizing the dynamic and multifactorial nature of microbiome remodeling (Schachtle and Rosshart, 2021).

#### 2.1.2 Immune system modulation and inflammaging

Aging is associated with profound changes in the immune system, a process referred to as immunosenescence, which plays a key role in the emergence of a chronic, low-grade inflammatory state known as inflammaging. This phenomenon involves the continuous activation of immune responses under sterile conditions, despite the absence of acute infection or tissue damage (Franceschi et al., 2017; Fulop et al., 2023).

The gut microbiota plays a central role in driving immune alterations associated with aging. This period is characterized by a reduction in beneficial commensal bacteria, such as *Bifidobacterium* and SCFA-producing taxa, alongside an expansion of pro-inflammatory Gram-negative bacteria, notably *Proteobacteria*. These microbial shifts disrupt the mucosal barrier, enabling microbes to reach from the gut lumen to other organs, build up toxins in the blood, and trigger an immune response (Badal et al., 2020; Singh A. et al., 2024).

Immunosenescence is characterized by significant alterations in both innate and adaptive immunity, including diminished neutrophil phagocytic function, altered monocyte cytokine secretion, accumulation of late-differentiated memory T Cells, reduced naïve T-cell populations, and a shift toward pro-inflammatory T-cell phenotypes (Fulop et al., 2023; Santoro et al., 2021). In contrast, successful aging may be supported by adaptive immunomodulatory mechanisms that mitigate inflammaging (Santoro et al., 2021).

At the mechanistic level, inflammaging is driven not only by external microbial stimuli but also by the intracellular accumulation of damaged self-molecules—commonly referred to as ‘molecular garbage’—such as mitochondrial DNA (mtDNA) and misfolded proteins, which can activate innate immune sensors, including inflammasomes (Franceschi et al., 2017). The ‘garb-aging’ hypothesis emphasizes the critical role of impaired proteostasis and defective autophagy in aging cells in sustaining chronic immune activation and inflammation. Furthermore, experimental studies provide strong evidence for the role of gut microbiota in immune aging. Transfer of microbiota from aged mice to young germ-free recipients has been shown to induce key features of inflammaging, including increased pro-inflammatory signaling, impaired gut barrier function, and systemic immune activation (Kim and Jazwinski, 2018; Thevaranjan et al., 2017).

In addition, dietary interventions such as adherence to the Mediterranean diet or caloric restriction have been shown to beneficially modulate gut microbiota composition, reduce systemic inflammation, and attenuate inflammaging in older individuals (Calder et al., 2022; Di Giosia et al., 2022). The complex interplay among gut dysbiosis, innate immune activation, mitochondrial dysfunction, and systemic inflammation highlights the potential of targeting the gut microbiota and immune metabolism as promising strategies to strengthen immune resilience and support healthy aging (Fulop et al., 2023; Singh A. et al., 2024).

#### 2.1.3 Intestinal barrier dysfunction and increased permeability

Age-related alterations in gut microbiota composition, particularly the decline of beneficial taxa such as *Bifidobacterium* and the overgrowth of facultative anaerobes and *Proteobacteria,* are closely linked to impaired intestinal barrier function. Aging is associated with the downregulation of key tight junction proteins, including claudins, occludins, and zonula occludens-1 (ZO-1), leading to increased epithelial permeability and promoting the systemic translocation of microbial products such as lipopolysaccharides (LPS; Gamez-Macias et al., 2024; Nicholson et al., 2012). LPS accumulation contributes to age-related low-grade inflammation, and aging-associated gut barrier dysfunction may further promote blood–brain barrier disruption, neuroinflammation, and cognitive decline (Gaber et al., 2024; Wu et al., 2021).

SCFAs, particularly butyrate, contribute to epithelial barrier integrity, whereas aging-related reductions in SCFA production may promote intestinal permeability and systemic inflammation (Liu et al., 2024; Zhu et al., 2024). Recent experimental studies further substantiate the causal relationship between gut dysbiosis and barrier dysfunction. FMT from aged donors has been shown to increase intestinal permeability, elevate systemic LPS levels, and enhance the production of inflammatory cytokines in young germ-free recipients (Zhang et al., 2022; Zhu et al., 2024). In contrast, colonization with microbiota from young donors restores gut barrier integrity and attenuates atrial NLRP3 inflammasome activation, ultimately reducing systemic inflammation and mitigating associated cardiac dysfunctions (Zhang et al., 2022).

Given these findings, probiotic supplementation has gained attention as a potential approach to mitigate aging-associated gut barrier dysfunction. Supplementation of human-origin probiotic formulations in aged mice have been demonstrated to upregulate tight junction proteins such as ZO-1 and occludin, lower circulating LPS levels, restore taurine metabolism, and alleviate systemic inflammation (Ahmadi et al., 2020). Furthermore, probiotic interventions beneficially modulate gut microbial composition by enriching SCFA-producing genera, thereby restoring both epithelial and BBB integrity and attenuating age-related neuroinflammation and cognitive decline (Yang et al., 2020).

### 2.2 Interactions between microbiota and brain function

#### 2.2.1 Microbial metabolites and neuroactive compounds

Microbial-derived metabolites, particularly SCFAs, are essential mediators of communication along the gut-brain axis. These metabolites help preserve gut barrier function, regulate the permeability of the BBB, support the maturation of microglial cells, and influence the production of neurotrophic factors such as brain-derived neurotrophic factor (BDNF; Cryan et al., 2019; Dandamudi et al., 2024). With aging, SCFA levels tend to decline, a change that has also been observed in individuals living with neuropsychiatric conditions such as depression. This decline points to a critical link between microbial metabolism and brain health, underscoring the importance of maintaining a healthy gut microbiota throughout the lifespan (Morkl et al., 2020; Nagpal et al., 2019).

Butyrate shows neuroprotective effects by regulating epigenetic mechanisms, including the inhibition of histone deacetylases and the upregulation of BDNF expression. These processes help promote synaptic plasticity and support memory formation (Nagpal et al., 2019). In germ-free animal models, the absence of SCFAs has been linked to impaired neurogenesis, abnormal dendritic structures, and disrupted neurotransmitter balance, highlighting the essential role of SCFA-producing bacteria in maintaining CNS function (Cryan et al., 2019).

In addition to SCFAs, gut microbiota also influences brain health through the regulation of tryptophan metabolism. Microbial modulation of this pathway shapes the balance between serotonin and kynurenine production. Dysregulation within this axis can reduce central serotonin levels while increasing neurotoxic kynurenine metabolites, contributing to stress vulnerability and accelerating neurodegenerative changes (Gao et al., 2024; Kolobaric et al., 2024).

Psychobiotic interventions targeting specific microbial strains offer a promising strategy for modulating the gut-brain axis. Recent experimental findings suggest that supplementation with *Lactococcus lactis* WHH2078 and *Lactococcus cremoris* WHH2080 in aged mice can help restore gut 5-hydroxytryptophan (5-HTP) levels, reduce hippocampal neuroinflammation, and improve both cognitive performance and emotional regulation (Gao et al., 2024).

Disruptions in gut microbial balance can also contribute to the production of neurotoxic metabolites such as palmitic amide, which further compromise intestinal and BBB integrity, fueling neuroinflammation and cognitive decline (Pan et al., 2023). Restoration of healthy microbiota through dietary interventions or FMT has been associated with the reductions in systemic inflammation and improvements in cognitive function during aging (Pan et al., 2023).

#### 2.2.2 Gut-brain signaling pathways: vagus nerve and HPA axis

The gut–brain axis relies on intricate, bidirectional communication pathways involving neural, endocrine, and immune signaling, with the vagus nerve and the hypothalamic–pituitary–adrenal (HPA) axis serving as key mediators. Among these, the vagus nerve provides the most direct neural link, transmitting sensory information from the gastrointestinal (GI) tract to the nucleus tractus solitarius (NTS) and onward to brain regions involved in regulating stress responses, appetite, mood, and cognitive functions (Peterson, 2020).

Vagal afferent fibers form close connections with enteroendocrine cells (EECs), creating a pathway through which microbial metabolites such as short-chain fatty acids (SCFAs), serotonin, and neuropeptides can send signals from the gut to the brain. These signals activate important pathways, including glutamatergic and serotonergic systems, allowing gut microbes to directly influence CNS functions like appetite control, energy balance, emotional regulation, and memory processing (Longo et al., 2023; Simao et al., 2023). Stimulation of the vagus nerve has been shown to promote hippocampal neurogenesis, increase BDNF expression, and improve cognitive functions, highlighting its essential role in supporting brain plasticity and cognitive health (Rusch et al., 2023). In contrast, damage to the vagus nerve, whether through surgical vagotomy, aging, or metabolic disorders, weakens gut-brain communication and may accelerate neurodegenerative changes (Wang et al., 2023).

Alongside vagal pathways, the hypothalamic–pituitary–adrenal (HPA) axis plays a central role in coordinating stress responses. Activation of the HPA axis triggers the release of corticotropin-releasing hormone (CRH), adrenocorticotropic hormone (ACTH), and cortisol, all of which can affect gut permeability and alter microbial composition. Increased gut permeability due to dysbiosis allows microbial components to enter the bloodstream, promoting systemic inflammation. This inflammatory state, in turn, activates the HPA axis, creating a cycle that further disrupts gut-brain communication (Toader et al., 2024).

Neuroactive molecules produced by gut microbes, including gamma-aminobutyric acid (GABA), serotonin, and tryptophan metabolites, play an important role in CNS function by influencing neurotransmission and supporting synaptic plasticity. Changes in these microbial pathways have been linked to stress resilience, mood regulation, and the preservation of cognitive functions (Peterson, 2020).

Emerging evidence suggests that disruptions in gut microbiota composition and impaired vagal signaling contribute to the development of Alzheimer’s disease (AD) by promoting neuroinflammation, weakening the BBB, and encouraging amyloid-beta deposition (Chen et al., 2021; Simao et al., 2023). Clinical studies have shown that reduced microbial diversity, along with an increase in pro-inflammatory bacteria such as some *Escherichia* species and *Shigella*, are early markers of cognitive decline, highlighting the gut-brain axis as a promising focus for strategies aimed at slowing neurodegenerative processes.

#### 2.2.3 Gut microbiota and cognitive aging

The gut microbiota has emerged as a key regulator of cognitive aging, influencing brain health through its effects on neuroinflammatory pathways, blood–brain barrier (BBB) integrity, and neuronal plasticity. Disruptions in microbial composition, particularly reductions in beneficial metabolites such as SCFAs, can impair microglial function and promote the accumulation of neurotoxic proteins, setting the stage for age-related cognitive decline (Loh et al., 2024). A randomized controlled trial demonstrated that supplementation with *Bifidobacterium bifidum* BGN4 and *Bifidobacterium longum* BORI for 12 weeks improved mental flexibility, increased serum BDNF levels, and reduced perceived stress in older adults. Notably, reductions in pro-inflammatory bacterial genera, such as *Eubacterium* and *Clostridiales*, were inversely associated with serum BDNF levels, suggesting a potential link between gut microbial modulation and enhanced cognitive resilience (Kim et al., 2021).

Microbial dysbiosis in aging is associated with increased gut permeability, systemic inflammation, and disruption of BBB integrity. Increased circulating levels of LPS and microbial-derived secondary bile acids have been detected in patients with Alzheimer’s disease, correlating with amyloid-*β* accumulation and cognitive impairment (Bradley and Haran, 2024). Notably, secondary bile acids of microbial origin have been identified within the brain tissue of individuals with Alzheimer’s disease, providing compelling evidence for the translocation of microbial metabolites from the gut to the brain.

In parallel, emerging studies highlight the contribution of dietary patterns and metabolic disorders to gut-brain dysfunction during aging. Obesity, high-fat diets, and the resulting metabolic inflammation exacerbate dysbiosis, further promoting neuroinflammation and accelerating cognitive decline (Leigh and Morris, 2020). On the other hand, modifiable lifestyle factors, such as nutritional interventions and physical activity, have demonstrated potential in restoring microbial diversity and promoting the production of anti-inflammatory metabolites, thereby contributing to the preservation of cognitive function and mitigating age-related neurocognitive decline (Melzer et al., 2021; Puri et al., 2023). Psychobiotic interventions targeting specific microbial strains, such as *Lactobacillus rhamnosus* GG, have also shown promise in improving cognitive performance among older adults with mild cognitive impairment, potentially through modulation of gut microbial composition and a reduction in pro-inflammatory taxa such as *Prevotella* (Aljumaah et al., 2022).

## 3 Recent evidence for protective effects of gerobiotics against neurodegenerative diseases

### 3.1 Animal studies

Recent preclinical animal studies have provided consistent evidence that gerobiotics exert beneficial physiological effects on neurodegenerative disease models (summarized in Table 1). Early findings demonstrated that administration of *Lactobacillus brevis* OW38 in aged mice alleviated inflammaging, lowered systemic and colonic pro-inflammatory cytokines, and reduced markers of cellular senescence, resulting in improved memory performance comparable to young mice (Jeong et al., 2016). In models of autoimmune encephalomyelitis resembling multiple sclerosis, mixtures of *Lactobacillus* and *Bifidobacterium* strains decreased disease severity, preserved myelin integrity, and enhanced neuromuscular function (Consonni et al., 2018). Several Alzheimer’s disease (AD) models confirmed cognitive improvements and structural brain protection with gerobiotic supplementation. A formula containing *Lactobacillus acidophilus, Lactobacillus casei*, *Bifidobacterium bifidum*, and *Bifidobacterium lactis* improved memory, synaptic integrity, and reduced glial activation in senescence-accelerated mice, alongside lower systemic inflammatory markers (Yang et al., 2020). Similarly, *Lactobacillus plantarum* and *Bifidobacterium longum* restored memory performance in infection- and LPS-induced cognitive impairment, accompanied by reduced neuroinflammation and increased BDNF levels (Lee et al., 2021).

**Table 1:** Animal studies on gerobiotics in neurodegenerative disease (2015–2025; a summary).

Reference	Year	Gerobiotic strain(s)	Subjects and procedures	Physiological effects	Mechanism of action
Jeong et al. (2016)	2016	*Lactobacillus brevis* OW38	Aged mice (18 months old) were given OW38 (10^9^ CFU) orally for 8 weeks; young adult mice as baseline controls.	Ameliorated inflammaging and age-related memory decline. improved working memory ↓colonic/serum LPS; ↓TNF-alpha, ↓IL-1β; Markers of cellular senescence (p16, p53) in colon and hippocampus were also suppressed	↓ NF-kB-mediated inflammation; restored gut barrier; ↑BDNF; ↑doublecortin; supporting hippocampal neurogenesis and cognitive function.
Consonni et al. (2018)	2018	*Lactobacillus casei* (LC) *and Lactobacillus rhamnosus* (LR)) + *Bifidobacterium breve* (BB) *and Bifidobacterium longum* (BL)	Lewis rat EAE model (autoimmune encephalomyelitis for MS); either LC + LR or BB + BL, at dose of 2 × 10^9^ CFU	↓ disease severity; ↑ neuromuscular function; preserved myelin	Immunoregulation via ↑ TGF-beta and regulatory T Cells Overall, the probiotics shifted gut and peripheral immunity toward regulatory responses, attenuating the CNS autoimmune attack.
Yang et al. (2020)	2020	“ProBiotic-4” mix (*B. lactis, B. bifidum, L. casei, L. acidophilus*)	Senescence-accelerated SAMP8 mice (aging/AD model); oral probiotic mix for 12 weeks	↑ memory and synaptic integrity; ↓ glial activation; ↓ IL-6, TNF-alpha, LPS; ↓ gut/BBB leakiness	Inhibited TLR4/NF-κB and RIG-I pathways in the brain, reducing neuroinflammation. Decreased oxidative DNA damage markers (*γ*-H2AX, 8-OHdG) and prevented RIG-I aggregation in neurons, indicating suppression of pro-inflammatory signaling.
Lee et al. (2021)	2021	*Lactobacillus plantarum* NK151 + *Bifidobacterium longum* NK173	C57BL/6 mice with cognitive impairment induced by *E. coli* K1 infection or systemic LPS. Daily oral gavage of each strain or a mix. Y-maze test (for working memory), novel object recognition (for recognition memory), to assess cognitive impairment.	↑ Y-maze and object recognition performance; ↓ IL-1β, IL-6, TNF-alpha; ↓ microglia; ↑ BDNF	↓ gut-derived LPS and NF-kB signaling; ↑ synaptic plasticity
Shamsipour et al. (2021)	2021	*Lactobacillus plantarum* + *Bifidobacterium bifidum*	Subjects: Male Wistar rats Intervention duration: 8 weeks. Groups (*n* = 8 per group): Control (healthy) Aβ (Alzheimer’s) Aβ + MIIT (moderate-intensity interval training) Aβ + Probiotic (*L. plantarum* + *B. bifidum*) Aβ + MIIT + Probiotic (combined treatment) Assessments: Passive avoidance test for memory, cresyl violet staining (hippocampal cell death), immunohistochemistry for ChAT, RT-PCR for BDNF expression.	↑ short-term memory; ↓ hippocampal cell death; ↑ ChAT and BDNF	Enhanced cholinergic activity and neurotrophic support (↑BDNF) in the brain, promoting neuroprotection.
Song et al. (2022)	2022	*Lactobacillus plantarum* DP189	Mice received D-galactose + aluminum to induce AD-like pathology, 10-week DP189 oral treatment Measurements/assessments: Behavioral tests for cognition. Neurotransmitter levels (serotonin, dopamine, GABA). Histological evaluation: neuronal damage; amyloid-β deposition; tau pathology. Gut microbiota composition Molecular signaling pathway: PI3K/Akt/GSK-3β pathway activity	↑ learning/memory; ↓ A-beta, tau hyperphosphorylation, neuronal damage; ↑ serotonin, dopamine, GABA.	Inhibited tau pathology via PI3K/Akt/GSK-3β signaling modulation, ultimately protecting neurons and cognitive function.
Pan et al. (2022)	2022	*Pediococcus pentosaceus* (GABA-producing probiotic)	MPTP-induced Parkinson’s in C57BL/6 mice; daily oral *P. pentosaceus* for 4 weeks	↑ motor function (better pole, rotation, beam-walk tests) ↓ dopaminergic loss and alpha-synuclein in the brain. Restored antioxidant defenses in the brain: elevated SOD1, GPx1, Nrf2 levels, and lowered oxidative stress markers.	↑ GABA/Nrf2 signaling, which upregulated the Nrf2 pathway in neurons (higher Nrf2, SOD1, GPx1; decreased Keap1 inhibitor). ↓ oxidative stress and dysbiosis.
Webberley et al. (2023)	2023	Lab4P consortium (*Lactobacillus* & *Bifidobacterium* mix)	3xTg-AD Alzheimer’s mice (with or without high-fat diet); probiotic supplementation for 12–24 weeks.	Preserved object recognition and hippocampal spine density	↓ hippocampal pro-inflammatory gene expression
Xin et al. (2024)	2024	LBE multistrain mix (*Lactobacillus acidophilus, Bifidobacterium longum, and Enterococcus faecalis*)	SOD1-G93A Amyotrophic lateral sclerosis (ALS) transgenic mice; daily oral gavage from age 60 days to end-stage.	Delayed progression; preserved motor neurons; ↓ SOD1 aggregation; improved gut structure	↓ TNF-alpha/IL-1β; ↑ SCFAs and autophagy.
Aktas et al. (2024)	2024	*Lacticaseibacillus rhamnosus* E9	MPTP mice, Oral supplementation with E9 for 15 days, 10^8^ CFU /day	↑ locomotion ↓ catalepsy, and improved performance in wire-hanging test; preserved TH/dopamine; ↑ ZO-1/occludin	Improved gut barrier and microbiota; ↓ ROS/neurodegeneration
Qiao et al. (2024)	2024	*Akkermansia muciniphila*	MPTP mice, 21 days oral supplementation	↑ motor function; ↓ dopaminergic loss; ↑ TH expression	↓ colonic inflammation/permeability; altered SCFAs; ↑ hippocampal neurogenesis
Prajapati et al. (2025)	2025	*Lactobacillus* + *Enterococcus* strains (infant-gut isolates, cocktail)	APP/PS1 transgenic Alzheimer’s mice; 10-strain probiotic (1 × 10^11^ CFU/day) in drinking water for 16 weeks	↑ cognition; ↓ Aβ, microglial activation, neuroinflammation; preserved BBB	Less inflammatory gut microbiome; strengthened gut/BBB tight junctions
Dong et al. (2025)	2025	*Lacticaseibacillus reuteri* (GABA-producing strain)	MPTP-induced Parkinson’s mouse model; oral *L. reuteri* given after microbiota depletion (antibiotics), compared to GABA supplementation.	Alleviated PD motor deficits and protected dopaminergic neurons (improved rotarod/traction performance, preserved tyrosine hydroxylase in substantia nigra). Supplementing GABA mimicked the probiotic’s neuroprotective effects.	*L. reuteri* → ↑ endogeneous GABA → activation of GABA receptor (GABRB1) → p-AKT ↑ → inhibited GSK3β → GPX4 up → ferroptosis suppressed → neuronal survival & improved motor function in PD model

AD, Alzheimer’s Disease; ALS, Amyotrophic Lateral Sclerosis; APP/PS1, Amyloid Precursor Protein / Presenilin 1 transgenic mice; Aβ, Amyloid-β; BBB, Blood–Brain Barrier; BDNF, Brain-Derived Neurotrophic Factor; ChAT, Choline Acetyltransferase; CFU, Colony Forming Unit; EAE, Experimental Autoimmune Encephalomyelitis; GABA, Gamma-Aminobutyric Acid; GPx / GPX4, Glutathione Peroxidase /Isoform 4; IL-1β / IL-6, Interleukin-1 beta / Interleukin-6; LPS, Lipopolysaccharide; MIIT, Moderate-Intensity Interval Training; MS-Multiple Sclerosis; MPTP, 1-Methyl-4-phenyl-1,2,3,6-tetrahydropyridine; NF-κB, Nuclear Factor kappa-light-chain-enhancer of activated B cells; Nrf2, Nuclear factor erythroid 2–related factor 2; ROS, Reactive Oxygen Species; SCFAs, Short-Chain Fatty Acids; SNpc, Substantia Nigra pars compacta; SOD1, Superoxide Dismutase 1; TH, Tyrosine Hydroxylase; TLR4, Toll-Like Receptor 4; TNF-α, Tumor Necrosis Factor-alpha; ZO-1, Zonula Occludens-1.

In a combined exercise and probiotic intervention, *L. plantarum* with *B. bifidum* improved short-term memory, decreased hippocampal neuronal loss, and elevated cholinergic activity, highlighting synergistic neuroprotection (Shamsipour et al., 2021). Moreover, *L. plantarum* DP189 administration attenuated amyloid-*β* accumulation, tau hyperphosphorylation, and neuronal damage, while increasing neurotransmitter levels, including serotonin, dopamine, and GABA, leading to marked improvements in learning and memory (Song et al., 2022). These effects were observed in *Lab4P* consortium-treated Alzheimer’s transgenic mice, where cognitive performance and hippocampal dendritic spine density were preserved even under metabolic stress (Webberley et al., 2023). Finally, an infant-gut derived probiotic cocktail protected APP/PS1 mice from amyloid pathology, reduced microglial activation, and maintained blood–brain barrier integrity, yielding significant cognitive gains (Prajapati et al., 2025).

Parkinson’s disease (PD) models also revealed strong physiological benefits of gerobiotics. *Pediococcus pentosaceus* improved motor performance, reduced dopaminergic neuron loss, and limited *α*-synuclein accumulation while restoring antioxidant defenses (Pan et al., 2022). Supplementation with *Lacticaseibacillus rhamnosus* E9 in MPTP mice alleviated locomotor deficits, preserved striatal dopamine, and enhanced intestinal barrier integrity (Aktas et al., 2024). Likewise, *Akkermansia muciniphila* improved motor functions, preserved dopaminergic neurons, and enhanced hippocampal neurogenesis in the same model (Qiao et al., 2024). Further, *L. reuteri* administration in PD mice protected against dopaminergic cell loss and improved motor coordination, paralleling the benefits of direct GABA supplementation (Dong et al., 2025). Beyond AD and PD, gerobiotic interventions showed promising effects in amyotrophic lateral sclerosis (ALS). In SOD1-G93A transgenic mice, a mixture of *Lactobacillus acidophilus*, *Bifidobacterium longum*, and *Enterococcus faecalis* prolonged survival, delayed disease progression, reduced pathological SOD1 aggregation, and preserved motor neuron integrity (Xin et al., 2024).

Overall, animal studies consistently indicate that gerobiotics enhance memory, motor function, and neuronal survival across multiple neurodegenerative disease models. These effects are accompanied by reductions in amyloid and tau pathology, preservation of neurotransmitter balance, strengthening of synaptic structures, and attenuation of neuroinflammation. Collectively, these findings highlight the therapeutic potential of gerobiotics in mitigating age- and disease-related neurodegeneration.

### 3.2 Human studies

Over the past decade, clinical investigations have increasingly explored the role of gerobiotics in neurodegenerative diseases, with outcomes spanning cognitive, metabolic, inflammatory, and gastrointestinal domains (summarized in Table 2). In Alzheimer’s disease (AD), Akbari et al. (2016) reported that a 12-week probiotic milk intervention was associated with higher Mini-Mental State Exam (MMSE) scores compared with placebo, together with reductions in systemic inflammation (hs-CRP) and oxidative stress (MDA), as well as improved insulin resistance indices (Akbari et al., 2016). Similarly, Tamtaji et al. (2019) found that probiotic and selenium co-supplementation was associated with a modest improvement in MMSE scores, accompanied by lower hs-CRP levels and enhanced total antioxidant capacity (Tamtaji et al., 2019). In contrast, Agahi et al. (2018) reported no significant changes in cognitive scores or inflammatory and oxidative markers in predominantly severe AD patients, suggesting that the effects of probiotic interventions may vary according to disease stage (Agahi et al., 2018). Leblhuber et al. (2018), in a short-term trial, observed decreases in fecal zonulin and increases in *Faecalibacterium prausnitzii*, suggesting improved gut barrier integrity, but no measurable cognitive benefit (Leblhuber et al., 2018). Ton et al. (2020), using kefir-based synbiotics, reported reductions in inflammatory cytokines, reactive oxygen species markers, and apoptosis rates in blood cells, together with potential improvements in selected cognitive domains. However, these findings should be interpreted cautiously given the limited sample size and heterogeneity of the available clinical evidence (Ton et al., 2020).

**Table 2:** Clinical studies on gerobiotics in neurodegenerative disease (2015–2025; a summary).

Reference	Year	Gerobiotic strains	Subjects and procedures	Physiological effects	Mechanisms of action
Akbari et al. (2016)	2016	*L.*acidophilus, *L. casei*, *L. fermentum*, *Bifidobacterium bifidum* (2 × 10^9^ CFU each)	60 elderly AD patients (60–95 years old) 12-week double-blind RCT (probiotic milk vs. milk placebo). Baseline and post-treatment cognitive tests (MMSE) and blood biomarkers measured.	Cognitive: MMSE score improved by ~28% in probiotic group vs. placebo. Metabolic: Significant reductions in inflammation (hs-CRP –17.6% vs. + 45% placebo) and oxidative stress (MDA − 22% vs. + 3% placebo); improved insulin metabolism (HOMA-IR + 29% vs. + 77% placebo).	Anti-inflammatory & antioxidant action: Probiotic intake attenuated systemic inflammation and lipid peroxidation, which may protect neurons. Proposed gut–brain-axis effects include reduced peripheral inflammation and oxidative stress, contributing to better cognitive function in AD.
Barichella et al. (2016)	2016	Fermented milk product with multiple probiotic strains (*Streptococcus salivarius subsp thermophilus, Enterococcus faecium, Lactobacillus rhamnosus* GG*, Lactobacillus acidophilus, Lactobacillus plantarum, Lactobacillus paracasei, Lactobacillus delbrueckii subsp bulgaricus, and Bifidobacterium (breve and animalis subsp lactis)*) + prebiotic fiber (inulin)	120 PD patients with chronic constipation, 4-week double-blind trial (2:1 randomization to probiotic-fiber fermented milk vs. placebo milk).	Stool consistency and straining improved modestly with the probiotic milk and no significant adverse events were noted. PD motor symptoms were not affected over this short duration.	Combined probiotic + fiber action: The multi-strain fermented milk plus inulin fiber acted as a synbiotic, enriching gut flora and increasing fecal bulk, thereby improving constipation in PD. Probiotic bacteria likely produced SCFAs from the fiber, which stimulate colonic motility and draw water into the bowel. By enhancing gut health (a gerobiotic effect on an aging gut), this intervention addresses a common PD non-motor issue. The improved GI function may also aid in better absorption of PD medications and overall comfort.
Inoue et al. (2018)	2018	*B. longum* BB536*, B. infantis* M-63*, B. breve* M-16 V *and B. breve* B-3 *(approximately 1.25 × 10^10^ CFU each)*	79 healthy elderly subjects (65–75 y); 12-week RCT; four arms: probiotic + exercise, exercise only, probiotic only, control.	Cognitive performance improved most in probiotic + exercise group; probiotic-only group improved bowel habits and showed modest cognitive benefit.	Gut microbiota balance and SCFA production improved; synergistic gut–brain–muscle axis effects.
Leblhuber et al. (2018)	2018	*Multi-strain (L. casei* W56*, L. acidophilus* W22*, L. paracasei* W20*, B. lactis* W52*, L. plantarum* W62*, L. salivarius* W24*, B. lactis* W51*, L. lactis* W19*)*	20 outpatients with Alzheimer’s dementia (9 females, 11 males), mean age. Probiotic supplementation for 4 weeks; laboratory tests of immune activation (serum neopterin), tryptophan breakdown (kynurenine / tryptophan ratio), gut inflammation markers, and fecal microbiota composition measured before and after intervention	Decrease in fecal zonulin concentration. Increase in *Faecalibacterium prausnitzii* abundance in fecal samples vs. baseline. Increase in serum kynurenine concentration. Correlation between changes in neopterin and changes in Kyn/Trp ratio	Modulation of gut microbiota composition leading to improved intestinal barrier function Altered tryptophan metabolism (increased kynurenine, changed Kyn/Trp ratio), implicating immune activation via macrophages/dendritic cells (neopterin correlation) Reduced gut-derived inflammation / leaky gut (lower zonulin), which may reduce systemic and neuro-inflammation in AD.
Agahi et al. (2018)	2018	*L. fermentum, L. plantarum, and B. lactis or L.acidophilus, B. bifidum, and B. longum The probiotic group received one of each capsule every other day, 3 × 10^9^ CFU*	AD patients, double-blind, randomized, placebo-controlled study over 12 weeks. Cognitive assessment, Biomarkers measured.	No statistically significant improvement in cognitive test scores in probiotic group vs. placebo in patients with severe AD. not significant changes in the inflammatory/oxidative biomarkers	Modifying gut microbiota could modulate inflammation/oxidative stress, which are implicated in AD. But severity of disease seemed to affect responsiveness: in severe AD, probiotic intervention had little effect, suggesting that once disease pathology is advanced, gut microbiota interventions may be less effective. Also, time of administration, dosage and probiotic strain formulation likely matter greatly. Early/mild disease might respond better.
Tamtaji et al. (2019)	2019	*L. acidophilus*, *B. bifidum*, *B. longum* (2 × 10^9^ CFU each) + selenium (200 μg/day) vs. selenium alone vs. placebo.	79 AD patients (55–100 y), 12-week double-blind trial (three arms). MMSE and metabolic/inflammatory markers assessed pre/post.	Cognitive: MMSE improved (+1.5 points) with probiotic+Se vs. minimal change in Se-only (+0.5) or placebo (−0.2; *p* < 0.001). Immune/Metabolic: Combined probiotic+Se markedly lowered serum hs-CRP (−1.6 mg/L vs. + 0.1 placebo, *p* < 0.001) and boosted antioxidant capacity (+89 vs. − 0.7 mmol/L, *p* = 0.001). Improvements in insulin sensitivity (QUICKI ↑, insulin ↓) and cholesterol profile were also seen.	Synergistic geroprotective effects: Probiotic + selenium co-supplementation reduced systemic inflammation and oxidative stress in AD. The probiotic strains likely modulated gut microbiota to produce antioxidants and anti-inflammatory metabolites, enhancing selenium’s effects. This gut–brain-axis modulation is thought to improve neuronal environment and cognitive outcomes in AD.
Kobayashi et al. (2019)	2019	*Bifidobacterium breve* A1 (Morinaga strain MCC1274)	27 older adults with MCI, 24-week open-label single-arm study (no placebo). Participants took *B. breve* A1 daily (two capsules containing approximately 2.0 × 10^10^ CFU)). Cognitive tests (MMSE, Digit Symbol Test) and mood/gut symptoms were tracked every 8 weeks.	Cognitive: Mean MMSE score increased at 24 weeks, indicating improved global cognition in MCI. Participants’ attention and processing speed (DSST) also showed upward trends. Mood/QoL: Depression and GI symptom scores significantly improved during probiotic treatment, suggesting better mood and gut health.	Gut–brain-axis enhancement: *B. breve* A1 reduces neuroinflammation via anti-inflammatory and neuroprotective metabolites from the probiotic. The strain may modulate microglial activation and gut-derived inflammation, thereby improving cognitive function and mood. Continued intake potentially helped gut microbiota in favor of neuroprotective profiles, reflected in better cognition and quality of life.
Hwang et al. (2019)	2019	*Lactobacillus plantarum* C29 *(DW2009, fermented soybean)*	100 participants with MCI, aged 55–85; 12-week multicenter RCT; daily intake of DW2009 vs. placebo; cognitive assessments with CERAD-K battery and serum biomarkers.	Significant improvement in verbal learning and memory scores in probiotic group vs. placebo.	Gut–brain axis modulation; systemic inflammation reduction; increased neurotrophic support (BDNF).
Ibrahim et al. (2020)	2020	Hexbio® multi-strain probiotic + FOS prebiotic (total 6 × 10^10^ CFU/day): *L. acidophilus*, *L. casei*, *L. lactis*, *B. bifidum*, *B. infantis*, *B. longum*	55 PD patients with chronic constipation (Rome III criteria), 8-week placebo-controlled RCT (probiotic-FOS granules vs. placebo fermented milk). Primary outcomes: bowel movement frequency (stool diaries), gut transit time (GTT) by radiopaque markers; secondary: PD non-motor symptoms (NMSS), quality of life (PDQ-39), UPDRS motor scores.	PD Symptoms: No significant differences in global PD non-motor symptom scores or motor UPDRS between groups over this short period benefits were confined to GI motility. Mild reversible side effects occurred in 4 probiotic patients.	By correcting dysbiosis (notably increasing beneficial bacteria) PD patients’ constipation was relieved. This gerobiotic effect on the gut did not translate to short-term motor changes, but it significantly improved constipation, an important non-motor PD symptom, via modulating the gut–brain axis (enteric nervous system activity and gut motility signals).
Xiao et al. (2020)	2020	*Bifidobacterium breve* MCC1274 *(strain A1), dosage: 2 × 10^10^ CFU/day*	80 older adults; 16-week double-blind RCT; probiotic vs. placebo; RBANS + MMSE.	Significant improvement in RBANS total score in probiotic vs. placebo Significant gains in RBANS scores: immediate memory, visuospatial/constructional ability, and delayed memory	Possible enhancement of gut-brain axis function; reducing low-grade inflammation may help improve cognition; Neuroprotective support possibly via increased production of beneficial metabolites or reduction of harmful ones by *B. breve* A1; balancing immune response and perhaps oxidative stress.
Ton et al. (2020)	2020	*Probiotic-fermented milk with kefir grains*	13 AD patients, 3 months, uncontrolled clinical investigation, Cognitive assessment, cytokine expression, systemic oxidative stress levels, and blood cell damage biomarkers	Improvement in cognitive performance on several classical cognitive tests: memory, visual–spatial / abstraction abilities, executive / language functions. Decrease in inflammatory cytokines and oxidative stress markers Increase in nitric oxide (NO) bioavailability. Improvements in serum protein oxidation, mitochondrial dysfunction, DNA damage / repair, and reduction in apoptosis in blood cells	The intervention may act as an adjuvant therapy by targeting three major aging / AD-related pathological axes: inflammation, oxidative damage, and cellular damage
Kim et al. (2021)	2021	*Bifidobacterium bifidum* BGN4 and *B. longum* BORI (probiotic capsule, total 2 × 10^10^ CFU/day)	63 community-dwelling seniors (≥65 y), 12-week multicenter RCT (placebo vs. multi-strain probiotic). Cognitive tests, life quality and mood scales, stress hormone, and serum BDNF levels were evaluated pre/post. Gut microbiome was analyzed via 16S rRNA sequencing.	Cognitive/Mood: Probiotic-treated elders showed significantly greater improvement in executive functioning (mental flexibility) compared to placebo. They also reported reduced perceived stress and improved life-satisfaction scores. No significant changes in memory or other domains were observed. Neurotrophic & Microbiota changes: The probiotic group had a rise in serum BDNF not seen in controls. Gut microbiota shifts included reduced abundance of pro-inflammatory genera (e.g., *Clostridiales*) after 12 weeks. Notably, changes in certain gut bacteria correlated inversely with BDNF levels (as BDNF ↑, pro-inflammatory taxa ↓).	Gut–brain modulation: The *Bifidobacterium* strains likely enhanced neurotrophic support (BDNF upregulation) and reduced gut inflammation, contributing to better cognitive flexibility. By decreasing inflammation-causing microbes and increasing beneficial metabolites, the gerobiotic regimen alleviated chronic stress and improved CNS plasticity in aging adults. This suggests a mechanism of enhanced brain-derived neurotrophic factor via microbiome changes, supporting cognitive and mood benefits in the elderly.
Asaoka et al. (2022)	2022	*Bifidobacterium breve* MCC1274 at 2 × 10^10^ CFU/day	MCI patients (65–88 y), 24-week multicenter RCT (55 probiotic vs. 60 placebo analyzed). Outcomes: Alzheimer’s Disease Assessment Scale–cognitive (ADAS-cog) and MMSE subscales, brain MRI for atrophy (VSRAD analysis), and gut microbiota profiling.	Cognitive: Overall ADAS-cog and total MMSE did not differ significantly vs. placebo, but specific domains improved—e.g., orientation (ADAS-cog subscore) was significantly better in the probiotic group by 24 weeks. In those with lower baseline cognition (MMSE <25), probiotic users had improved orientation in time and writing ability vs. placebo Neuroimaging: Placebo group showed progression of brain atrophy over 6 months, whereas probiotic supplementation suppressed the advance of atrophy, especially in participants with more pronounced baseline atrophy. Gut microbiota composition showed no major overall shifts.	Neuroprotective and anti-atrophy effect: Probiotic *B. breve* A1 may act by reducing neuroinflammation or toxic metabolites, thereby slowing cerebral atrophy. The preserved brain volume in the probiotic group suggests gerobiotic action on fundamental aging processes in the brain. Even without large microbiota changes, the strain might modulate gut-derived tryptophan or immune signaling to protect neurons. Thus, gerobiotic *B. breve* A1 potentially improves specific cognitive functions and mitigates brain structural decline in MCI.
Ghalandari et al. (2023)	2023	Comflor® multi-strain capsule (4.5 × 10^11^ CFU/day total): *L. plantarum*, *L. casei*, *L. acidophilus*, *L. bulgaricus*, *B. infantis*, *B. longum*, *B. breve*, *Streptococcus thermophilus*	30 PD patients (≥60 y) with functional constipation, 8-week randomized placebo-controlled trial (1 capsule/day probiotic vs. maltodextrin/starch placebo). All patients continued standard PD meds; bowel habits, laxative use, and UPDRS motor scores were tracked.	Motor Symptoms: No significant difference in UPDRS motor scores between probiotic vs. placebo after 8 weeks (motor function remained stable in both groups).	Although 8 weeks was too short to affect neurodegeneration, relieving constipation improves patients’ quality of life and may secondarily reduce gut-derived toxin absorption, potentially benefiting long-term PD outcomes.
Ramadan et al. (2025)	2025	Synbiotic (probiotic + prebiotic): *Lactobacillus acidophilus* (two sachets/day alongside standard PD meds)	66 PD patients, 3-month randomized trial: all received Levodopa/Carbidopa; one group additionally took a daily synbiotic (2 sachets). Outcomes: UPDRS (Parts I–IV) and blood biomarkers (TNF-α, MDA, BDNF, α-synuclein) pre vs. post.	Motor & Non-motor Symptoms: After 12 weeks, the synbiotic group had greater improvements in UPDRS scores than the control (meds-only) group. Motor function, activities of daily living, and non-motor symptoms all improved more with the synbiotic. Biomarkers: The synbiotic significantly lowered serum TNF-α and MDA compared to control, while increasing BDNF. α-synuclein levels showed a favorable trend (reduced aggregation) in the probiotic group.	Neuroinflammation reduction & neurotrophic support: By targeting the gut–brain axis, the synbiotic exhibited promising neuroprotective effects, likely through dampening neuroinflammatory pathways. Lower TNF-α suggests reduced microglial activation, and higher BDNF indicates enhanced neuronal survival and plasticity. The probiotic components presumably improved gut permeability and microbial composition, thereby decreasing peripheral inflammation that can worsen PD, while prebiotic fibers fuel beneficial bacteria. This holistic gerobiotic approach modulates immune signals and oxidative stress, translating into both motor and non-motor symptom relief in PD patients.

AD, Alzheimer’s Disease; MCI, Mild Cognitive Impairment; PD, Parkinson’s Disease; MS, Multiple Sclerosis; RCT, Randomized Controlled Trial; MMSE, Mini-Mental State Exam; UPDRS, Unified Parkinson’s Disease Rating Scale; ADAS-cog-Alzheimer’s Disease Assessment Scale, Cognitive Subscale; hs-CRP, high-sensitivity C-reactive protein; MDA, malondialdehyde; BDNF, brain-derived neurotrophic factor; SCFA, short-chain fatty acids; EDSS, Expanded Disability Status Scale; DASS, Depression Anxiety Stress Scales; QUICKI- quantitative insulin sensitivity check index; NO, Nitric Oxide; SCFA, Short-Chain Fatty Acids; RCT, Randomized Controlled Trial; CFU, Colony-Forming Unit; CERAD-K, Consortium to Establish a Registry for Alzheimer’s Disease-Korean version (neuropsychological test battery); RBANS, Repeatable Battery for the Assessment of Neuropsychological Status; MRI, Magnetic Resonance Imaging.

For Mild Cognitive Impairment (MCI), results were more consistent. Hwang et al. (2019) reported significant improvements in verbal learning and memory after 12 weeks of *Lactobacillus plantarum* C29-fermented soybean (Hwang et al., 2019). Kobayashi et al. (2019) demonstrated increases in MMSE scores (+1.7 points at 24 weeks) and reductions in depression and gastrointestinal symptoms with *Bifidobacterium breve* A1 (Kobayashi et al., 2019). Xiao et al. (2020) further confirmed these effects, with Repeatable Battery for the Assessment of Neuropsychological Status (RBANS) total scores rising significantly in the probiotic group, particularly in immediate and delayed memory and visuospatial domains (Xiao et al., 2020). Asaoka et al. (2022) extended these findings, showing that although global ADAS-cog scores did not differ, orientation and writing ability improved in subgroups with lower baseline cognition, and importantly, Magnetic Resonance Imaging (MRI) analysis suggested attenuation of brain atrophy in the probiotic group (Asaoka et al., 2022).

In healthy older adults, Inoue et al. (2018) observed the greatest cognitive gains when bifidobacteria supplementation was combined with resistance exercise, with additional benefits for bowel habits (Inoue et al., 2018). Kim et al. found that supplementation with *Bifidobacterium bifidum* BGN4 and *B. longum* BORI improved executive functioning, increased serum BDNF levels, reduced perceived stress, and was associated with a decline in pro-inflammatory gut taxa (Kim et al., 2021).

In PD, clinical outcomes focused predominantly on gastrointestinal symptoms. Barichella et al. (2016) showed that a fermented milk synbiotic increased weekly complete bowel movements significantly (Barichella et al., 2016). Ibrahim et al. (2020) confirmed similar findings, with probiotic supplementation reducing whole gut transit time and improving stool consistency (Ibrahim et al., 2020). Ghalandari et al. (2023) also demonstrated higher bowel movement frequency and improved stool form compared with placebo (Ghalandari et al., 2023). While motor scores (Unified Parkinson’s Disease Rating Scale-UPDRS) were not significantly improved in these short trials, Ramadan et al. (2025) provided new evidence that synbiotic supplementation alongside standard dopaminergic therapy significantly improved both motor and non-motor UPDRS subscales, while lowering serum TNF-*α* and MDA, and increasing BDNF levels, indicating both symptomatic and biomarker improvements (Ramadan et al., 2025).

In human studies, these findings show that gerobiotics may improve cognitive outcomes in MCI, enhance systemic metabolic and inflammatory profiles in early-to-moderate AD, and ameliorate constipation and bowel function in PD, with emerging evidence for motor and non-motor improvements when combined with synbiotics. The heterogeneity of results in AD suggests that disease stage and intervention duration are critical determinants of responsiveness.

## 4 The mechanisms of action of gerobiotics on neurodegenerative diseases

The mechanisms of action of gerobiotics in neurodegenerative diseases are summarized in Table 1 (animal studies) and Table 2 (human studies). Based on these findings, the proposed mechanisms can be categorized under the following main headings: neurotrophic pathways, antioxidant and anti-inflammatory mechanisms, and BBB integrity. However, the presence of some metabolites, immunological factors, etc. can be overlapping factors, indicated by biomarkers.

### 4.1 Neurotrophic and neurotransmitter pathways

Gerobiotics influence neuronal communication and plasticity by modulating neurotrophic factors and neurotransmitters. In AD animal models, *L. plantarum* combined with *B. bifidum* elevated ChAT activity and BDNF expression, which preserved cholinergic function and promoted hippocampal cell survival (Shamsipour et al., 2021). *L. plantarum* DP189 further boosted dopamine, serotonin, and GABA levels, while preventing amyloid-*β* accumulation and tau phosphorylation (Song et al., 2022). In PD models, *Pediococcus pentosaceus* and *L. reuteri* increased GABA production, protected dopaminergic neurons, and improved locomotor performance via GABA receptor–AKT signaling, suggesting neurotransmitter restoration as a core pathway (Dong et al., 2025; Pan et al., 2022).

It has been shown that probiotic interventions increased serum BDNF in older adults, which correlated with enhanced executive function and reduced stress (Kim et al., 2021). In MCI patients, *B. breve A1* and *L. plantarum* C29 improved verbal learning, orientation, and memory scores, with MRI evidence showing slower brain atrophy progression (Asaoka et al., 2022; Hwang et al., 2019). These results highlight the role of gerobiotics in sustaining neuronal plasticity and cognitive resilience through neurotransmitter and neurotrophic modulation.

### 4.2 Antioxidant and anti-inflammatory mechanisms

Neuroinflammation contributes to neuronal loss and cognitive decline. Gerobiotics downregulate NF-κB and TLR4 signaling, reducing pro-inflammatory cytokines (IL-1*β*, IL-6, TNF-*α*) and limiting microglial overactivation (Lee et al., 2021). In autoimmune encephalomyelitis models, combinations of *Lactobacillus* and *Bifidobacterium* expanded T-regulatory cells, induced tolerogenic dendritic cells, and increased systemic TGF-β, thereby attenuating CNS inflammation and demyelination (Consonni et al., 2018).

In clinical studies, AD patients showed reduced hs-CRP and systemic inflammatory markers after probiotic supplementation (Akbari et al., 2016; Tamtaji et al., 2019). Kefir-based synbiotics lowered inflammatory cytokines and oxidative stress markers while improving multiple cognitive domains (Ton et al., 2020). In PD patients, synbiotics significantly decreased serum TNF-*α* and oxidative stress (MDA), while increasing BDNF, supporting both anti-inflammatory and neurotrophic outcomes (Ramadan et al., 2025). This indicates that gerobiotics modulate peripheral and central immune activity, dampening chronic inflammation that drives neurodegeneration.

Mitochondrial dysfunction and ROS accumulation are central in AD and PD. Gerobiotics alleviate oxidative stress by activating Nrf2-dependent antioxidant pathways, upregulating SOD1, GPx, and GPX4, and lowering Keap1 inhibition (Pan et al., 2022). In PD models, *L. reuteri* suppressed ferroptosis via the AKT–GSK3β–GPX4 axis, maintaining dopaminergic neuron survival (Dong et al., 2025). In AD models, probiotics reduced oxidative DNA damage, indicated by the decrease of markers (*γ*-H2AX, 8-OHdG), protecting neurons against mitochondrial dysfunction (Yang et al., 2020). AD patients receiving probiotic milk exhibited significant reductions in MDA (−22% vs. placebo +3%) and improved insulin sensitivity indices (Akbari et al., 2016). Synbiotic interventions with probiotics and selenium further enhanced antioxidant capacity (+89 mmol/L) and decreased oxidative stress while improving metabolic parameters (Tamtaji et al., 2019). These findings suggest that mitochondrial stabilization and oxidative stress reduction are conserved gerobiotic mechanisms across models and clinical trials.

### 4.3 Gut–brain axis: barrier integrity and autophagy

Disruption of gut and BBB integrity is strongly implicated in neurodegeneration. In animal studies, *L. brevis* OW38 and *L. rhamnosus* E9 restored intestinal tight junction proteins (ZO-1, Occludin) and suppressed gut-derived endotoxin leakage, thereby reducing systemic inflammation and brain senescence markers (Aktas et al., 2024; Jeong et al., 2016). In AD models, probiotic cocktails, containing 5 *Enterococcus* and 5 *Lactobacillus* strains, preserved BBB integrity, preventing neuroinflammation and amyloid pathology (Prajapati et al., 2025).

Clinically, reduced fecal zonulin and increased abundance of *Faecalibacterium prausnitzii* after probiotic supplementation indicated improved gut barrier integrity (Leblhuber et al., 2018). These barrier-enhancing effects are particularly relevant for PD, where constipation and dysbiosis exacerbate systemic and neuronal inflammation. Gerobiotics therefore act at the interface of gut–brain communication, stabilizing barrier functions that otherwise accelerate disease progression.

Microbial metabolites are key mediators of the gut–brain axis. Gerobiotics increase SCFAs such as butyrate, propionate, and isovaleric acid, which enhance hippocampal neurogenesis, reduce inflammation, and modulate microglial activity (Qiao et al., 2024). In ALS models, probiotic supplementation boosted SCFA production and enhanced autophagy, facilitating clearance of misfolded SOD1 proteins and preserving motor neurons (Xin et al., 2024).

In AD and PD, gerobiotics activated autophagic pathways to degrade pathological aggregates, including amyloid-β, tau, and α-synuclein (Dong et al., 2025; Song et al., 2022). These processes slowed neurodegenerative pathology and improved cognitive and motor outcomes. By coupling microbial metabolite regulation with autophagy, gerobiotics address one of the most fundamental drivers of neurodegeneration: toxic protein accumulation.

Human studies provide translational evidence of mechanistic pathways. In AD, probiotic and synbiotic supplementation improved MMSE scores, decreased hs-CRP and MDA, and improved insulin resistance indices (HOMA-IR, QUICKI; Akbari et al., 2016, Tamtaji et al., 2019). In MCI, *B. breve* and *L. plantarum* supplementation led to significant improvements in RBANS and ADAS-cog subscores, with concomitant increases in serum BDNF and reduced depressive symptoms (Asaoka et al., 2022; Kobayashi et al., 2019; Xiao et al., 2020). In PD, most trials initially showed improvements in bowel movement frequency and gut transit time (Barichella et al., 2016; Ghalandari et al., 2023; Ibrahim et al., 2020). However, more recent evidence demonstrated that synbiotics not only improved gastrointestinal function but also enhanced motor and non-motor UPDRS scores, reduced inflammation (indicated by the decrease in pro-inflammatory biomarkers TNF-α, MDA), and it also increased BDNF (Ramadan et al., 2025). Collectively, these biomarker modulations confirm the clinical translation of gerobiotic mechanisms into measurable health outcomes in patients.

## 5 Potential adverse effects of gerobiotics

Theoretically, influencing the microecosystem with respect to potential inflammations and the inflammatory response manifested through the effects of the immune system, combined with the biological variability, can lead to multifarious outcomes. Furthermore, the variability in microbial strains, dosages, treatment durations, and study populations poses a significant challenge to the development of standardized therapeutic protocols. Potential paradoxical harmful effects of inadequate gerobiotics treatment should be avoided (Gyriki et al., 2025).

Rao et al. reported evidence that *Lactobacillus* and *Streptococcus* sp. are capable of colonizing the small bowels, producing D-lactic acid and ultimately causing Small Intestinal Bacterial Overgrowth (SIBO), ultimately impairing mental conditions (Rao et al., 2018). Furthermore, a case report highlighted that probiotics are capable of provoking D-lactic acidosis in a patient with the rare complication of short bowel syndrome (Leung et al., 2025). Also, butyrate (derived from the aforementioned species) may act as a double-edged sword by exerting a suppressive effect on stem cells during injury, through FOXO3 activation, slowing down the cell cycle to reduce potentially genotoxic damage from oxidative stress, but also slowing down wound healing, potentially exacerbating inflammatory bowel diseases (Kaiko et al., 2016).

## 6 Limitations and future directions

Despite the growing body of evidence supporting the neuroprotective potential of gerobiotics, several limitations should be acknowledged. Considerable heterogeneity exists across studies in terms of probiotic strains, dosages, intervention durations, and study populations, which limits the comparability and generalizability of findings. In addition, most available evidence is derived from preclinical models, while human studies remain relatively limited and often involve small sample sizes. Additionally, there is a need for further mechanistic insights, particularly about the long-term effects and strain-specific actions of gerobiotics in humans. Also, potential paradoxical harmful effects of inadequate gerobiotics treatment should be avoided.

From a translational perspective, several challenges must still be addressed, including the standardization of formulations, interindividual variability in microbiome composition, uncertainty regarding strain-specific effects, and the limited availability of long-term clinical data. Future research should place greater emphasis on well-designed, large-scale randomized controlled trials to better define the efficacy, optimal dosage, and long-term safety of gerobiotics across different populations. Greater standardization of formulations and study protocols will also be necessary to improve reproducibility and facilitate clinical translation. At the same time, further mechanistic studies are needed to clarify the complex biological interactions within the microbiota–gut–brain axis, particularly through the use of integrated multi-omics approaches such as metagenomics, metabolomics, and transcriptomics. Special attention should be directed toward identifying strain-specific effects and elucidating how gerobiotics influence neuroinflammation, blood–brain barrier integrity, and key pathways involved in neurodegeneration. Addressing these challenges will be essential for translating the current body of experimental evidence into clinically meaningful approaches for the prevention and management of age-related neurodegenerative diseases.

## 7 Conclusion

The current results highlight the potential of gerobiotics as a promising intervention to support cognitive health and mitigate age-related neurodegenerative processes. The results of both preclinical and clinical studies indicate the potential of specific probiotic and postbiotic formulations to impact the microbiota–gut–brain axis, thereby reducing neuroinflammation, enhancing synaptic function, balancing immune responses, and restoring BBB integrity, ultimately improving cognitive performance. These effects appear to be particularly relevant during the early stages of neurodegenerative conditions and among individuals with mild cognitive impairment. Overall, gerobiotics represent a multifaceted and biologically plausible approach targeting key mechanisms involved in aging and neurodegeneration, with potential as an adjunctive strategy alongside existing therapeutic interventions. Furthermore, these findings may support the development of targeted microbiome-based interventions aimed at improving therapeutic efficacy and potentially reducing the required dosage of conventional pharmacological treatments.
